# Identification of Serum Circulating MicroRNAs as Novel Diagnostic Biomarkers of Gastric Cancer

**DOI:** 10.3389/fgene.2020.591515

**Published:** 2021-02-01

**Authors:** Yunjin Yao, Yongfeng Ding, Yuntong Bai, Quan Zhou, Hyun Lee, Xiawei Li, Lisong Teng

**Affiliations:** ^1^Department of Surgical Oncology, The First Affiliated Hospital, College of Medicine, Zhejiang University, Hangzhou, China; ^2^Department of Medical Oncology, The First Affiliated Hospital, College of Medicine, Zhejiang University, Hangzhou, China; ^3^Biomedical Engineering Department of Tulane University, New Orleans, LA, United States; ^4^Brown University, Warren Alpert School of Medicine, Providence, RI, United States; ^5^Department of Surgery, The Second Affiliated Hospital, Zhejiang University School of Medicine, Hangzhou, China

**Keywords:** gastric cancer, biomarkers, microRNAs, gene expression omnibus datasets, differential expression analysis

## Abstract

Gastric cancer (GC) is one of the leading causes of cancer-associated deaths worldwide. Due to the lack of typical symptoms and effective biomarkers for non-invasive screening, most patients develop advanced-stage GC by the time of diagnosis. Circulating microRNA (miRNA)-based panels have been reported as a promising tool for the screening of certain types of cancers. In this study, we performed differential expression analysis of miRNA profiles of plasma samples obtained from gastric cancer and non-cancer patients using two independent Gene Expression Omnibus (GEO) datasets: GSE113486 and GSE124158. We identified three miRNAs, hsa-miR-320a, hsa-miR-1260b, and hsa-miR-6515-5p, to distinguish gastric cancer cases from non-cancer controls. The three miRNAs showed an area under the curve (AUC) over 0.95 with high specificity (>93.0%) and sensitivity (>85.0%) in both the discovery datasets. In addition, we further validated these three miRNAs in two external datasets: GSE106817 [sensitivity: hsa-miR-320a (99.1%), hsa-miR-1260b (97.4%), and hsa-miR-6515-5p (92.2%); specificity: hsa-miR-320a (88.8%), hsa-miR-1260b (89.6%), and hsa-miR-6515-5p (88.7%); and AUC: hsa-miR-320a (96.3%), hsa-miR-1260b (97.4%), and hsa-miR-6515-5p (94.6%)] and GSE112264 [sensitivity: hsa-miR-320a (100.0%), hsa-miR-1260b (98.0%), and hsa-miR-6515.5p (98.0%); specificity: hsa-miR-320a (100.0%), hsa-miR-1260b (100.0%), and hsa-miR-6515.5p (92.7%); and AUC: hsa-miR-320a (1.000), hsa-miR-1260b (1.000), and hsa-miR-6515-5p (0.988)]. On the basis of these findings, the three miRNAs can be used as potential biomarkers for gastric cancer screening, which can provide patients with a higher chance of curative resection and longer survival.

## Introduction

Gastric cancer (GC) is the fifth most common cancer and the third leading cause of malignancy-related deaths worldwide with over 1 million annual incidences and approximately 783,000 associated deaths (Bray et al., 2018). The cancer stage at the time of diagnosis is an important prognostic factor. GC patients when diagnosed at an advanced stage had a 5-year survival rate less than 30% (Hundahl et al., 2000), while those diagnosed at an early stage had a 5-year survival rate of 70–90% (Okada et al., 2012; Choi et al., 2015). Thus, implementation of an effective screening method that allows for the detection of GC at its early stages is critical for higher favorable outcomes.

According to the current National Comprehensive Cancer Network guidelines, upper endoscopy is the most common screening approach for GC (Ajani et al., 2016; Japanese Gastric Cancer Association, 2017). Although it is a reliable method with a sensitivity of approximately 70–92% (Dooley et al., 1984; Hamashima et al., 2008; Choi et al., 2012), it is not the most preferred method due to its invasiveness, cost, and anesthetic requirements. In contrast, radiography is another available option for GC screening that bypasses the problems associated with upper endoscopy, but is not very reliable as its negative predictive value can be as high as 50% with a sensitivity as low as 14% (Dooley et al., 1984; Longo et al., 1989). Therefore, a GC screening modality with acceptable sensitivity is required for effective detection of GC at earlier stages.

The use of serum tumor markers has been proposed as an alternative method. Conventional markers, such as prostate-specific antigen (PSA), carbohydrate antigen 199 (CA199), and alpha-fetoprotein (AFP) are useful for screening prostate carcinoma, pancreatic carcinoma, and hepatic carcinoma, respectively (Sturgeon, 2002). A systemic review reported that the conventional tumor markers are not very effective as a screening tool for GC despite their association with tumor stage and patient survival (Shimada et al., 2014). Some of the alternative biomarkers, such as serum pepsinogen and trefoil factor 3, have been proposed, but neither their sensitivity nor specificity exceeded 80% (Miki, 2006; Aikou et al., 2011). Therefore, these biomarkers can be used as a screening test for high-risk subjects with atrophic gastritis, but not as a tool for screening cancer (Miki, 2006). In conclusion, neither classic tumor markers nor functional protein levels in the serum are ideal screening biomarkers for the primary diagnosis of GC.

MicroRNAs (miRNAs) are small non-coding regulatory RNAs (17–25 nucleotides) that to bind complementary sequences in the 3-untranslated regions (3-UTR) of various target mRNAs to promote degradation or translational repression (Caldas and Brenton, 2005). miRNAs play a role in almost all aspects of cancer biology, such as proliferation, apoptosis, invasion/metastasis, and angiogenesis (Lee and Dutta, 2009). In addition, miRNAs have been proposed as potential biomarkers for the diagnosis of several different cancer types, such as testicular germ cell tumors (miRNA-371a-3p: sensitivity 90.1% and specificity 94.0%) (Dieckmann et al., 2019), bladder cancer (7-miRNA panel: sensitivity 95% and specificity 87%) (Usuba et al., 2018), and hepatocellular cancer (miR-424: sensitivity 95.12% and specificity 87.13%) (Lin et al., 2015). Some studies also reported that several miRNAs have a potential value as diagnostic biomarkers of gastric cancer (Zhou et al., 2010; Cui et al., 2013; Su et al., 2014). However, most miRNA biomarkers have not been developed by comprehensive data mining based on miRNA profiling and even lack appropriate external validation of their efficacy (Link and Kupcinskas, 2018; Wei et al., 2019).

In the present study, we compared the miRNA expression profile in the plasma of gastric cancer patients with that of healthy controls using two discovery datasets to identify several miRNAs with promising performance. Then, we validated the diagnostic values of these miRNAs using two independent external datasets. Therefore, we propose the following three miRNAs, namely, hsa-miR-320a, hsa-miR-1260b, and hsa-miR-6151.5p that could act as potential diagnostic biomarkers of gastric cancer.

## Materials and Methods

### Study Cohort

To identify a robust circulating miRNA biomarker, we searched the Gene Expression Omnibus (GEO) database with specific keywords, namely (“stomach neoplasms” [MeSH Terms] OR gastric cancer [All Fields]) AND “Homo sapiens” [porgn] AND ((“microRNAs” [MeSH Terms] OR miRNA [All Fields]) AND (“blood” [Subheading] OR “blood” [MeSH Terms] OR blood [All Fields])). Then, four datasets using the same platform with a larger sample size were included: GSE113486, GSE124158, GSE112264, and GSE106817 (235 gastric cancer patients and 3,174 non-cancer controls in total) for further analysis. GSE113486 and GSE124158 (70 gastric cancer patients and 374 non-cancer controls) were used as the initial discovery cohort and the others (165 gastric cancer patients and 2,800 non-cancer controls) were used for independent validation. All the datasets were serum miRNA profiles based on the same microarray platform, 3D-Gene Huma miRNA V21_1.0.0. Data were downloaded using the Bioconductor package “GEOquery” in R and then normalized. The probe set IDs were converted to the official name of the miRNAs according to the annotation “GPL21263” in GEO. The clinical information we obtained from phenoData of corresponding datasets are summarized in Supplementary Table 4.

### Initial miRNA Signature Identification

**FIGURE 1:**
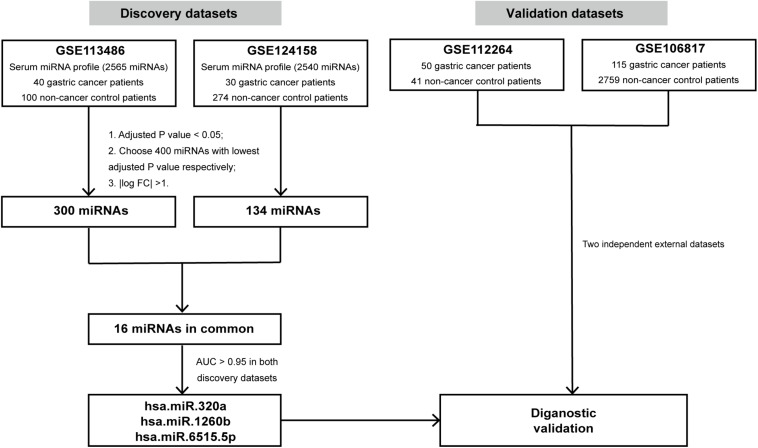
Study design. The schematic diagram represents the strategy for *in silico* discovery and validation of the miRNA signatures. AUC, area under the curve.

The workflow for identifying miRNA biomarkers is displayed in Figure 1. First, we compared the expression profiles between gastric cancer patients and non-cancer controls using the R package “Limma” in the discovery cohorts (GSE113486 and GSE124158, respectively). Two sets of differentially expressed (DE) miRNAs were then obtained. The selection criteria for the most significant DE miRNAs were as follows: (1) calculate the adjusted p value using the empirical Bayes method and choose those with *p* < 0.05; (2) choose 400 miRNAs with the lowest adjusted *p*-value; (3) calculate | log2 fold change| and choose those with | log2 fold change| >1. Further, the most significant DE miRNAs in both the discovery cohorts were identified as the candidate miRNAs for GC diagnosis. To evaluate the diagnostic capacity of the candidate miRNAs, hierarchical unsupervised clustering analysis was conducted on all candidate miRNAs and the receiver operating characteristic (ROC) curve analysis was performed for each candidate miRNA in both the discovery cohorts. The candidate miRNAs with an area under the curve (AUC) over 0.95 in both the discovery cohorts were selected.

### *In silico* Validation

To verify the robustness of the selected miRNAs, GSE112264 and GSE106817 were used as independent external datasets to distinguish gastric cancer samples from non-cancer controls. ROC curve analysis was performed and the AUC, sensitivity, and specificity were calculated for performance evaluation.

### Statistical Analysis

Statistical analysis was performed using R version 3.5.0. The DE analysis was performed using the “Limma” package in R, which is based on the empirical Bayes method and the linear models. The power calculations for the ROC curves were based on the one-sided DeLong’s test using the R package “pROC” (version 1.10.0). Unsupervised hierarchical clustering of DE miRNAs was analyzed using the “complete linkage” method based on Euclidean distance using the “pheatmap” package in R.

## Results

### Identification of Candidate Serum miRNAs for Gastric Cancer Detection

To discover biomarkers for gastric cancer, GSE113486 (40 gastric cancer patients and 100 non-cancer controls) and GSE124158 (30 gastric cancer patients and 274 non-cancer controls) datasets were downloaded from GEO as discovery datasets. The expression levels of all 2,565 miRNAs from GSE113486 and 2,540 miRNAs from GSE124158 were analyzed to identify DE miRNAs as the candidate biomarkers using the “limma” package in R. The complete profile of DE miRNAs in the two discovery datasets are shown in Volcano plots (Supplementary Figure 1). Then, 300 miRNAs and 134 miRNAs from GSE113486 and GSE124158, respectively (details in Supplementary Table 1) were identified as the most significant DE miRNAs (based on the screening criteria mentioned in section “Materials and Methods”).

**FIGURE 2:**
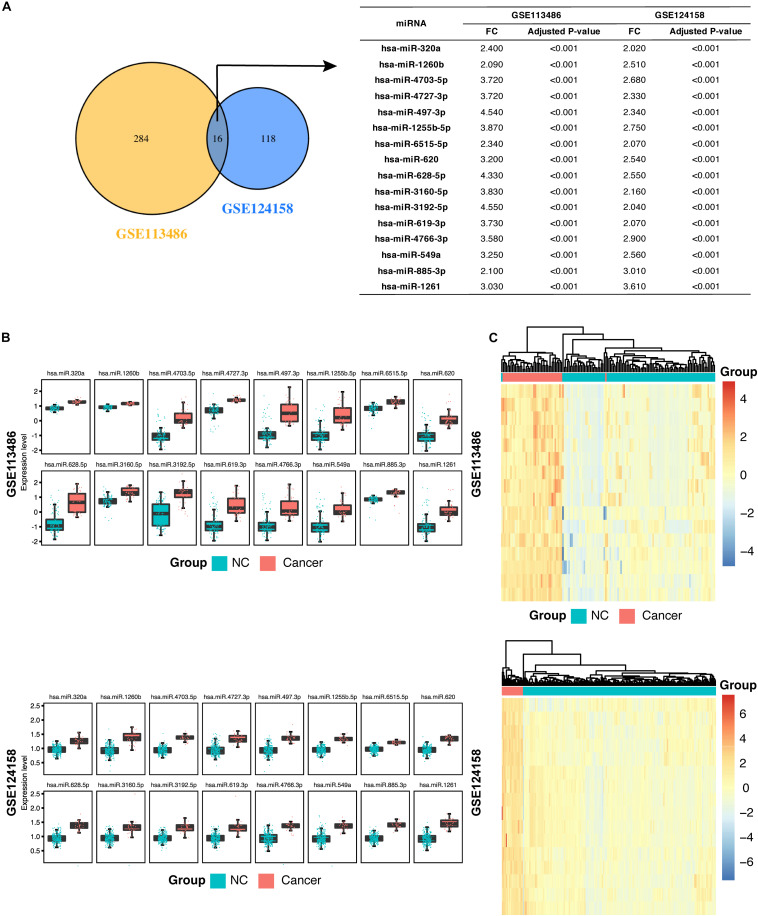
Identification of 16 candidate serum miRNA signatures and their characterization in the discovery datasets. **(A)** Venn diagram showing significant differentially expressed (DE) miRNAs in the discovery dataset GSE113458 and GSE124158. The 16 miRNAs are listed in the table on the right along with the adjusted *p*-value and fold change. **(B)** Box plots showing higher expression levels of the 16 identified miRNAs in gastric cancer patients compared with the non-cancer control patients in GSE113486 and GSE124158. **(C)** Heatmap showing a promising result of the hierarchical clustering analysis using the 16 identified miRNAs to distinguish different samples in GSE113486 and GSE124158. NC, non-cancer control patients.

In addition, as shown in Figure 2A, we found that 16 miRNAs (hsa-miR-320a, hsa-miR-1260b, hsa-miR-4703-5p, hsa-miR-4727-3p, hsa-miR-497-3p, hsa-miR-1255b-5p, hsa-miR-6515-5p, hsa-miR-620, hsa-miR-628-5p, hsa-miR-3160-5p, hsa-miR-3192-5p, hsa-miR-619-3p, hsa-miR-4766-3p, hsa-miR-549a, hsa-miR-885-3p, and hsa-miR-1261) were identified in both the discovery datasets and were defined as the candidate miRNAs for GC diagnosis. The fold change and adjusted p values of these 16 candidate miRNAs are listed in Figure 2A. We then examined the expression levels of these 16 candidate miRNAs in the two discovery datasets and demonstrated the values using a boxplot (Figure 2B). Although the expression levels of specific miRNAs in the two datasets differed, the GC patients expressed higher levels of the miRNAs compared to the non-cancer controls.

Next, we examined the overall diagnostic capacity of these candidate miRNAs in GC. A hierarchical unsupervised clustering analysis was performed to create a clear distinction between GC patient group and non-cancer control group. We found that samples in the discovery datasets could be distinguished into cancer and non-cancer control groups with only two misplaced samples in GSE113486. In addition, the heatmap showed some differences between samples in each group. In GSE113486, the samples in the middle of the figure show a significantly low miRNA expression level (blue color) on the heatmap compared with that in the GC group. However, miRNA expression levels of the samples on the right side of the figure are closer to that of the GC group (Figure 2C).

### Diagnostic Performance of Each miRNA to Further Filter the Candidate miRNAs

**FIGURE 3:**
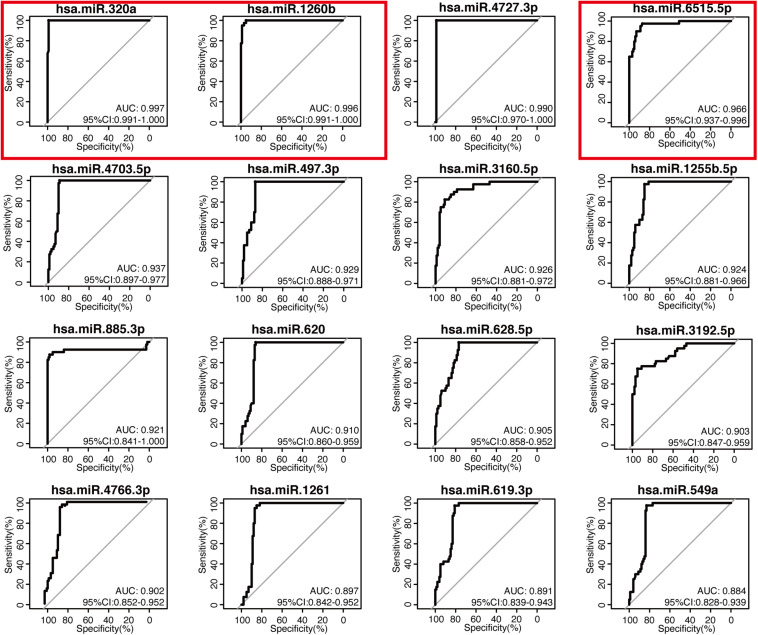
Diagnostic performance of the 16 candidate serum miRNA signatures in the discovery dataset GSE113486. The expression levels of 16 candidate miRNAs were individually analyzed using the receiver operating characteristic curve analysis in the GSE113486 cohort. The area under the curve (AUC), specificity, and sensitivity are calculated and displayed for the evaluation of the candidate miRNAs in the diagnosis of gastric cancer.

**FIGURE 4:**
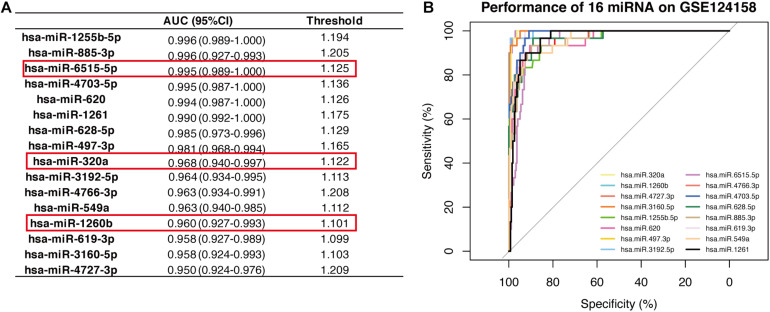
Diagnostic performance of the 16 candidate serum miRNA signatures in the discovery dataset GSE124158. **(A)** Shows the diagnostic performance of different miRNAs, wherein the threshold is the cut-off that decides whether a sample is cancerous or not. **(B)** Displays the overlaid receiver operating characteristic curves (ROC) of 16 individual miRNAs.

The 16-miRNA panel showed superior diagnostic capacity in gastric cancer. Next, we explored the possibility to further reduce the number of candidate miRNAs. Therefore, the diagnostic capacity of each miRNA among the 16 candidates was evaluated for both the discovery data sets by ROC analysis. We found that hsa-miR-320a (AUC 0.997, sensitivity 100.0%, and specificity 99.0%), hsa-miR-1260b (AUC 0.996, sensitivity 100.0%, and specificity 95.0%), hsa-miR-4727.3p (AUC 0.990, sensitivity 100.0%, and specificity 99.0%), and hsa-miR-6515.5p (AUC 0.966, sensitivity 97.5%, and specificity 87.0%) had better performance (all AUC values >0.95, maximum of AUC values are 1.00) in diagnosis for the discovery dataset, GSE113486 (Figure 3). Interestingly, in the other dataset, all 16 miRNAs, except for hsa-miR-4727-4p, had an AUC > 0.95 (Figure 4). Overall, hsa-miR-320a, hsa-miR-1260b, and hsa-miR-6515-5p had a robust performance with an AUC over 0.95 and high specificity and sensitivity in both the discovery datasets.

### Validation of Diagnostic Performance of the Three Selected miRNAs in External Datasets

**FIGURE 5:**
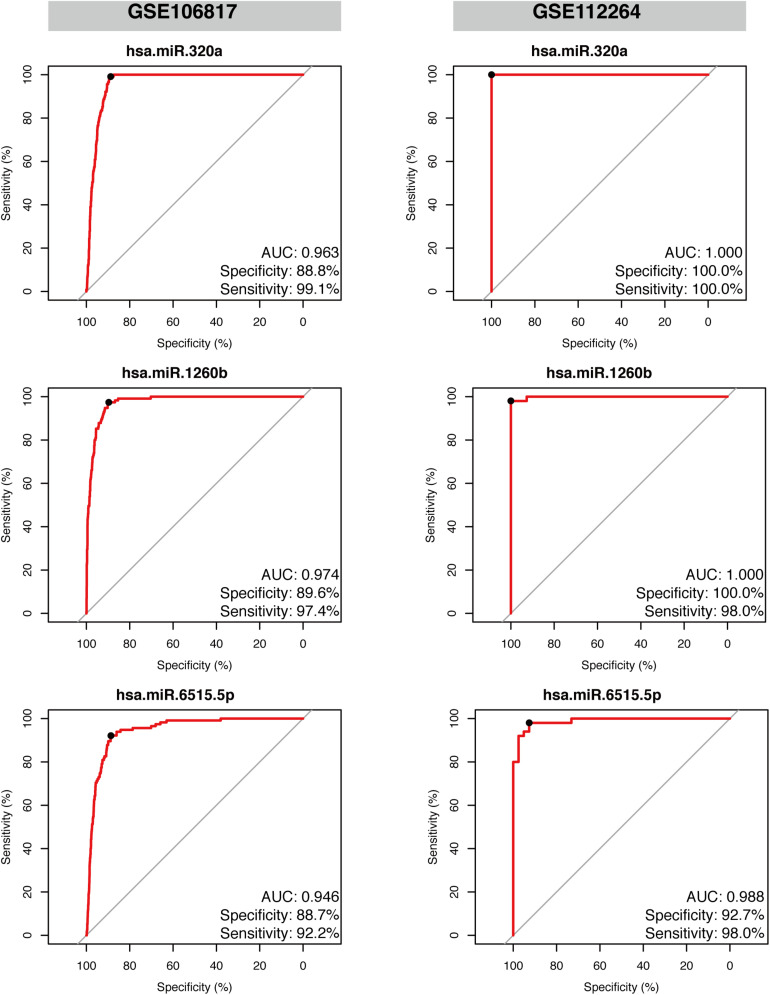
Validation of the diagnostic performance of the three selected miRNA signatures in two independent external datasets. Receiver operating characteristic curve (ROC) analysis was conducted on the two validation datasets: GSE106817 and GSE112264. Area under the curve (AUC), specificity, and sensitivity are calculated and displayed for each dataset.

Given the robust performance of hsa-miR-320a, hsa-miR-1260b, and hsa-miR-6515-5p in both the discovery datasets, we further examined their reliability in two independent external validation datasets, GSE112264 and GSE106817. As displayed in Figure 5, we found that all the three miRNAs had high performance and could efficiently distinguish the gastric cancer samples from non-cancer controls. The values were as follows: hsa-miR-320a had an AUC 0.963, sensitivity 99.1%, and specificity 88.8% in GSE106817 and an AUC 1.000, sensitivity 100.0%, and specificity 100.0% in GSE112264; hsa-miR-1260b had an AUC 0.974, sensitivity 97.4%, and specificity 89.6% in GSE106817 and an AUC 1.000, sensitivity 98.0%, and specificity 100.0% in GSE112264; and hsa-miR-6515.5p had an AUC 0.946, sensitivity 92.2%, and specificity 88.7% in GSE106817 and an AUC 0.988, sensitivity 98.0%, and specificity 92.7% in GSE112264.

## Discussion

In the early stages, GC is often asymptomatic or present with only non-specific symptoms. Intervention at this stage makes GC almost curable and thus, early detection and diagnosis are crucial to reduce the morbidity and mortality of GC. Although upper endoscopy remains the gold standard for detection of gastric cancer, the procedure is invasive and expensive and not universally acceptable as an ideal screening method, thus making it inconvenient compared with blood tests. This in turn delays the diagnostic process due to lack of screening. Therefore, in this study, we used a simple but effective strategy and identified hsa-miR-320a, hsa-miR-1260b, and hsa-miR-6515.5p as potential biomarkers for primary diagnosis of GC. Furthermore, hsa-miR-320a and hsa-miR-1260b was more stable in all the discovery and validation datasets.

Application of miRNAs for early detection of various types of cancer is a promising approach. Dieckmann et al. (2019) reported that microRNA-371a-3p could act as a new diagnostic biomarker for testicular germ cell tumors because it had a sensitivity and specificity of over 90%, which outperformed the classic markers such as alpha-fetoprotein, beta-human chorionic gonadotropin, and lactate dehydrogenase. Lin et al. (2015) conducted a similar study on hepatocellular carcinoma and identified a miRNA classifier containing seven miRNAs (miR-29a, miR-29c, miR-133a, miR-143, miR-145, miR-192, and miR-505). This classifier had a larger AUC than AFP in identifying small-sized tumors or early stage hepatocellular carcinoma and could detect α-fetoprotein-negative hepatocellular carcinoma in patients at risk.

However, current studies have certain drawbacks as pointed out in a review by Link and Kupcinskas (2018) after reviewing 75 original studies that analyzed the expression of miRNAs in blood/serum/plasma of the GC patients and the controls. First, most of the studies only focused on several miRNAs that they were interested in rather than a comprehensive miRNA profile (Link and Kupcinskas, 2018). Second, most of these studies had a smaller number of GC samples (5–40) and a non-cancer control samples (5–190). In addition, the outcomes of external validations in studies were relatively biased because the AUC, specificity, and sensitivity were reported with a 1:1 proportion of GC patients to controls, while in real-life settings, the proportion is close to 1:100–1,000 (Link and Kupcinskas, 2018). Hence, we tried to avoid these defects in the present study. First, the expression of miRNAs in two discovery sets was obtained from the same microarray platform: 3D-Gene Human miRNA V21_1.0.0, which presents the miRNA profile of the cohort and makes the original data more comprehensive and reliable. Second, our discovery and validation datasets had larger sample sizes with one of our validation datasets (GSE106817) containing 115 gastric cancer patients vs. 2,759 non-cancer controls, which mimics the proportion in real-life settings. In addition, the performance of miRNAs identified in our study is promising. Shin et al. (2015) reported a three-miRNA panel including miR-627, miR-629, and miR-652 with a remarkable sensitivity of 86.7% and specificity of 85.5% (123 GC vs. 111 non-cancer controls). A meta-analysis including 77 studies reported pooled outcomes of sensitivity, specificity, and AUC being 76, 81%, and 0.86/1.00, respectively (Wei et al., 2019). In the present study, the three identified miRNAs showed an AUC of about 0.95, in both the discovery and validation datasets with high sensitivity (>90%) and specificity (>88%). Interestingly, we found that high performance was achieved even when one miRNA was analyzed, thus making the screening process simpler and suitable. A combination of any two or three of the three identified miRNAs did not outperform any individual miRNA (Supplementary Table 2).

However, there are certain limitations to our study. First, detailed pathological information, such as the tumor node metastasis (TNM) stage, was not available because the cohorts analyzed in our study were derived from public databases. The incorporation of GC stage in the public datasets could be advantageous. Second, we only included gastric cancer patients and non-cancer controls in our study and did not evaluate the performance of these miRNAs to distinguish GC from other cancers. Therefore, future studies should focus on the identification of new biomarkers from early GC patients with confirmed pathology and include samples from patients with different types of cancers in the cohort. In addition, despite the high performance of these three miRNAs in our study, prospective studies based on high-throughput and multi-centered cohorts are required for further validation.

## Conclusion

In conclusion, this study provides a simple but effective strategy to identify highly sensitive and specific miRNAs for GC detection. Using this strategy, we identified three specific miRNAs: hsa-miR-320a, hsa-miR-1260b, and hsa-miR-6515-5p that had significantly high expression in the serum samples of gastric cancer patients and robust performance in distinguishing gastric cancer patients from the non-cancer controls.

## Data Availability Statement

The datasets analyzed for this study can be found in the Gene Expression Omnibus (https://www.ncbi.nlm.nih.gov/geo/), accession numbers GSE113486, GSE124158, GSE112264, and GSE106817.

## Author Contributions

The research project was designed by YY and YD and organized by LT. Data analysis was designed by YY and YD, conducted by YY. The first draft of the article was written by YY and YD, and the article was reviewed by QZ, HL, XL, and LT. All authors contributed to the article and approved the submitted version.

## Conflict of Interest

The authors declare that the research was conducted in the absence of any commercial or financial relationships that could be construed as a potential conflict of interest.
