# Supplementary material for: Identification of Serum Circulating MicroRNAs as Novel Diagnostic Biomarkers of Gastric Cancer
Source: Front Genet. 2021 Feb 1;11:591515. doi: 10.3389/fgene.2020.591515 (PMC7882724; doi:10.3389/fgene.2020.591515)
Supplement: Supplementary file 1 [file Data_Sheet_1.docx]

Supplementary Material

# Supplementary Figures and Tables

## Supplementary Figures


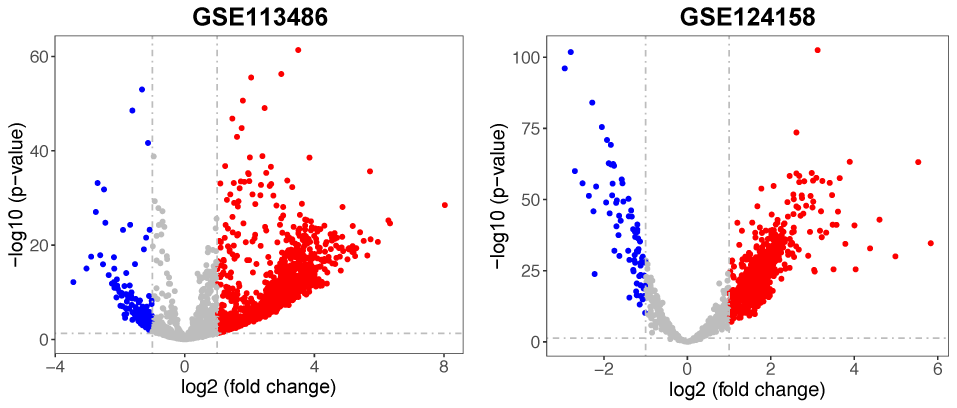


**Supplementary Figure 1.**Volcano plots showing differentially expressed miRNAs in two different discovery datasets (GSE113486 and GSE124158). MicroRNAs with adjusted P value < 0.05 and log_2_FC > 1 were defined as significantly overexpressed miRNAs, which were showed in red; meanwhile, those with adjusted P value < 0.05 and log_2_FC < -1 were defined as significantly under-expressed miRNAs, which were showed in blue. The other miRNAs were showed in grey.

## SupplementaryTable

| **Table1 Top significantly DE miRNAs in GSE113486** | | | | |
| --- | --- | --- | --- | --- |
| hsa-miR-1343-3p | hsa-miR-8073 | hsa-miR-6787-5p | hsa-miR-4787-3p | hsa-miR-320a |
| hsa-miR-6746-5p | hsa-miR-5100 | hsa-miR-8059 | hsa-miR-6131 | hsa-miR-4429 |
| hsa-miR-614 | hsa-miR-3184-5p | hsa-miR-1307-3p | hsa-miR-4286 | hsa-miR-4783-3p |
| hsa-miR-4732-5p | hsa-miR-4730 | hsa-miR-1260b | hsa-miR-4532 | hsa-miR-320b |
| hsa-miR-6717-5p | hsa-miR-296-3p | hsa-miR-1246 | hsa-miR-4454 | hsa-miR-23b-3p |
| hsa-miR-920 | hsa-miR-575 | hsa-miR-548ah-5p | hsa-miR-668-5p | hsa-miR-92a-3p |
| hsa-miR-1258 | hsa-miR-4708-3p | hsa-miR-29b-1-5p | hsa-miR-4536-3p | hsa-miR-1203 |
| hsa-miR-191-5p | hsa-miR-342-5p | hsa-miR-4703-5p | hsa-miR-4719 | hsa-miR-151a-5p |
| hsa-miR-4740-5p | hsa-miR-6511b-5p | hsa-miR-8060 | hsa-miR-4464 | hsa-miR-3924 |
| hsa-miR-3607-5p | hsa-miR-4711-5p | hsa-miR-4727-3p | hsa-miR-4477b | hsa-miR-4635 |
| hsa-miR-3153 | hsa-miR-2467-3p | hsa-miR-29b-3p | hsa-miR-3686 | hsa-miR-22-3p |
| hsa-miR-8076 | hsa-miR-23a-3p | hsa-miR-4525 | hsa-miR-7977 | hsa-miR-3674 |
| hsa-miR-4705 | hsa-miR-1277-3p | hsa-miR-208b-5p | hsa-miR-4515 | hsa-miR-5591-5p |
| hsa-miR-3194-3p | hsa-miR-570-5p | hsa-miR-4774-5p | hsa-miR-497-3p | hsa-miR-4419b |
| hsa-miR-759 | hsa-miR-664a-5p | hsa-miR-4795-5p | hsa-miR-103a-3p | hsa-miR-4259 |
| hsa-miR-1262 | hsa-miR-654-5p | hsa-miR-875-5p | hsa-miR-4764-5p | hsa-miR-3118 |
| hsa-miR-6501-3p | hsa-miR-1290 | hsa-miR-6765-3p | hsa-miR-4696 | hsa-miR-451a |
| hsa-miR-1255b-5p | hsa-miR-627-3p | hsa-miR-513c-3p | hsa-miR-3658 | hsa-miR-92b-3p |
| hsa-miR-651-3p | hsa-miR-376c-5p | hsa-miR-3136-3p | hsa-miR-3908 | hsa-miR-135b-3p |
| hsa-miR-3606-5p | hsa-miR-3607-3p | hsa-miR-4317 | hsa-miR-3923 | hsa-miR-6515-5p |
| hsa-miR-3688-5p | hsa-miR-561-5p | hsa-miR-605-3p | hsa-miR-124-3p | hsa-miR-4668-5p |
| hsa-miR-4718 | hsa-miR-4652-5p | hsa-miR-4666a-5p | hsa-miR-379-5p | hsa-miR-130a-3p |
| hsa-miR-17-3p | hsa-miR-5585-5p | hsa-miR-4771 | hsa-miR-4782-5p | hsa-miR-548av-5p |
| hsa-miR-620 | hsa-miR-1537-5p | hsa-miR-3179 | hsa-miR-3662 | hsa-miR-606 |
| hsa-miR-4490 | hsa-miR-653-3p | hsa-miR-548bb-3p | hsa-miR-628-5p | hsa-miR-4480 |
| hsa-miR-4256 | hsa-miR-4757-5p | hsa-miR-3912-3p | hsa-miR-3934-5p | hsa-miR-3611 |
| hsa-miR-561-3p | hsa-miR-6512-5p | hsa-miR-302f | hsa-miR-3121-3p | hsa-miR-548n |
| hsa-miR-4775 | hsa-miR-4662a-5p | hsa-miR-3613-5p | hsa-miR-891b | hsa-miR-548ad-3p |
| hsa-miR-4648 | hsa-miR-6073 | hsa-miR-4693-3p | hsa-miR-5579-3p | hsa-miR-5579-5p |
| hsa-miR-4755-3p | hsa-miR-3927-3p | hsa-miR-3117-5p | hsa-miR-5586-3p | hsa-miR-125a-3p |
| hsa-miR-3121-5p | hsa-miR-3609 | hsa-miR-7975 | hsa-miR-4779 | hsa-miR-3683 |
| hsa-miR-4276 | hsa-miR-4678 | hsa-miR-3160-5p | hsa-miR-3672 | hsa-miR-320e |
| hsa-miR-3671 | hsa-miR-514a-3p | hsa-miR-4742-5p | hsa-miR-4457 | hsa-miR-302e |
| hsa-miR-4536-5p | hsa-miR-510-5p | hsa-miR-151b | hsa-miR-24-3p | hsa-miR-3166 |
| hsa-miR-4272 | hsa-miR-4744 | hsa-miR-5587-5p | hsa-miR-374a-3p | hsa-miR-4693-5p |
| hsa-miR-4482-5p | hsa-miR-522-3p | hsa-miR-221-5p | hsa-miR-4999-3p | hsa-miR-3156-5p |
| hsa-miR-3192-5p | hsa-miR-3115 | hsa-miR-548au-5p | hsa-miR-4777-3p | hsa-miR-1245b-3p |
| hsa-miR-3119 | hsa-miR-3146 | hsa-miR-6822-5p | hsa-miR-4423-5p | hsa-miR-548i |
| hsa-miR-134-3p | hsa-miR-619-3p | hsa-miR-148b-5p | hsa-miR-4766-3p | hsa-miR-4448 |
| hsa-miR-556-5p | hsa-miR-539-3p | hsa-miR-548w | hsa-miR-889-3p | hsa-miR-4720-5p |
| hsa-miR-6760-5p | hsa-miR-6839-3p | hsa-miR-4691-3p | hsa-miR-5681a | hsa-miR-208a-3p |
| hsa-miR-5586-5p | hsa-miR-320d | hsa-miR-519a-3p | hsa-miR-5197-3p | hsa-miR-3684 |
| hsa-miR-549a | hsa-miR-1244 | hsa-miR-3606-3p | hsa-miR-3128 | hsa-miR-650 |
| hsa-miR-4804-5p | hsa-miR-4781-3p | hsa-miR-3910 | hsa-miR-551b-5p | hsa-miR-4753-5p |
| hsa-miR-1271-3p | hsa-miR-4679 | hsa-miR-4481 | hsa-miR-588 | hsa-miR-6788-3p |
| hsa-miR-3133 | hsa-miR-924 | hsa-miR-499a-5p | hsa-miR-4663 | hsa-miR-455-5p |
| hsa-miR-888-3p | hsa-miR-6838-3p | hsa-miR-4770 | hsa-miR-8061 | hsa-miR-5706 |
| hsa-miR-548u | hsa-miR-376b-5p | hsa-miR-5000-5p | hsa-miR-140-3p | hsa-miR-3668 |
| hsa-miR-545-3p | hsa-miR-5092 | hsa-miR-548al | hsa-miR-6866-5p | hsa-miR-4434 |
| hsa-miR-141-3p | hsa-miR-548y | hsa-miR-6755-5p | hsa-miR-1264 | hsa-miR-548ba |
| hsa-miR-5682 | hsa-miR-548aw | hsa-miR-3165 | hsa-miR-548h-5p | hsa-miR-4255 |
| hsa-miR-7-5p | hsa-miR-576-3p | hsa-miR-922 | hsa-miR-3689a-5p | hsa-miR-107 |
| hsa-miR-4677-3p | hsa-miR-885-3p | hsa-miR-4796-3p | hsa-miR-302c-5p | hsa-miR-548m |
| hsa-miR-4753-3p | hsa-miR-6739-5p | hsa-miR-618 | hsa-miR-3143 | hsa-miR-5002-5p |
| hsa-miR-1243 | hsa-miR-145-3p | hsa-miR-519d-5p | hsa-miR-143-3p | hsa-miR-10b-3p |
| hsa-miR-4494 | hsa-miR-548ad-5p | hsa-miR-519a-5p | hsa-miR-548ag | hsa-miR-4789-3p |
| hsa-miR-664b-5p | hsa-miR-888-5p | hsa-miR-1261 | hsa-miR-548p | hsa-miR-30b-3p |
| hsa-miR-320c | hsa-miR-2115-3p | hsa-miR-4710 | hsa-miR-3939 | hsa-miR-4500 |
| hsa-miR-587 | hsa-miR-5590-3p | hsa-miR-4477a | hsa-miR-5047 | hsa-miR-532-5p |
| hsa-miR-4299 | hsa-miR-548d-5p | hsa-miR-4504 | hsa-miR-4461 | hsa-miR-4444 |

| **Table 2 Top significantly DE miRNAs in GSE124158** | | | | |
| --- | --- | --- | --- | --- |
| hsa-miR-6862-3p | hsa-miR-4655-5p | hsa-miR-518a-3p | hsa-miR-380-5p | hsa-miR-591 |
| hsa-miR-1229-5p | hsa-miR-6885-3p | hsa-miR-1976 | hsa-miR-4458 | hsa-miR-93-5p |
| hsa-miR-7704 | hsa-miR-3149 | hsa-miR-4760-3p | hsa-miR-1295a | hsa-miR-216a-3p |
| hsa-miR-885-3p | hsa-miR-4463 | hsa-miR-186-3p | hsa-miR-4703-5p | hsa-miR-4666b |
| hsa-miR-4647 | hsa-miR-4708-5p | hsa-miR-3943 | hsa-miR-620 | hsa-miR-147a |
| hsa-miR-7155-5p | hsa-miR-571 | hsa-miR-3660 | hsa-miR-1255b-5p | hsa-miR-18b-3p |
| hsa-miR-6786-5p | hsa-miR-3170 | hsa-miR-181c-5p | hsa-miR-619-5p | hsa-miR-8065 |
| hsa-miR-6506-3p | hsa-miR-602 | hsa-miR-1181 | hsa-miR-1261 | hsa-miR-335-5p |
| hsa-miR-3186-5p | hsa-miR-6780b-3p | hsa-miR-1205 | hsa-miR-3192-3p | hsa-miR-361-5p |
| hsa-miR-4701-5p | hsa-miR-3666 | hsa-miR-873-5p | hsa-miR-3177-5p | hsa-miR-7150 |
| hsa-miR-137 | hsa-miR-3180 | hsa-miR-3180-3p | hsa-miR-628-5p | hsa-miR-4524b-5p |
| hsa-miR-6740-5p | hsa-miR-1304-5p | hsa-miR-934 | hsa-miR-4786-5p | hsa-miR-181d-5p |
| hsa-miR-4327 | hsa-miR-4765 | hsa-miR-5194 | hsa-miR-24-1-5p | hsa-miR-3646 |
| hsa-miR-8057 | hsa-miR-4748 | hsa-miR-5582-5p | hsa-miR-518c-5p | hsa-miR-1469 |
| hsa-miR-4267 | hsa-miR-6802-3p | hsa-miR-3192-5p | hsa-miR-497-3p | hsa-miR-617 |
| hsa-miR-4632-3p | hsa-miR-548j-3p | hsa-miR-3150b-5p | hsa-miR-501-5p | hsa-miR-1260b |
| hsa-miR-192-5p | hsa-miR-621 | hsa-miR-627-5p | hsa-miR-655-3p | hsa-miR-6515-5p |
| hsa-miR-4524b-3p | hsa-miR-4254 | hsa-miR-608 | hsa-miR-637 | hsa-miR-215-5p |
| hsa-miR-3186-3p | hsa-miR-511-3p | hsa-miR-3678-5p | hsa-miR-3161 | hsa-miR-518d-3p |
| hsa-miR-320a | hsa-miR-3978 | hsa-miR-4781-5p | hsa-miR-890 | hsa-miR-4535 |
| hsa-miR-4640-3p | hsa-miR-4675 | hsa-miR-302a-5p | hsa-miR-619-3p | hsa-miR-4310 |
| hsa-miR-548ax | hsa-miR-921 | hsa-miR-3160-5p | hsa-miR-4715-5p | hsa-miR-2110 |
| hsa-miR-363-5p | hsa-miR-550a-3-5p | hsa-miR-4653-5p | hsa-miR-146b-5p | hsa-miR-4524a-3p |
| hsa-miR-4766-3p | hsa-let-7e-3p | hsa-miR-549a | hsa-miR-3152-3p | hsa-miR-214-3p |
| hsa-miR-625-3p | hsa-miR-3150b-3p | hsa-miR-6083 | hsa-miR-3911 | hsa-miR-3191-3p |
| hsa-miR-3154 | hsa-miR-6125 | hsa-miR-6126 | hsa-miR-3155a | hsa-miR-1199-5p |
| hsa-miR-6087 | hsa-miR-4727-3p | hsa-miR-4761-5p | hsa-miR-4685-5p |  |

**Supplementary Table1&2.**List of 300 miRNAs and 134 miRNAs identified as top significantly DE miRNAs from GSE113486 and GSE124158, respectively

| **Table 3 Performance of combinations** | | | | | | | | | |
| --- | --- | --- | --- | --- | --- | --- | --- | --- | --- |
|  | **GSE124158** | | | **GSE112264** | | | **GSE106817** | | |
| **miRNACombination** | **AUC** | **Sensitivity** | **Specificity** | **AUC** | **Sensitivity** | **Specificity** | **AUC** | **Sensitivity** | **Specificity** |
| hsa-miR-320a + hsa-miR-1260b | 0.928 | 96.7% | 88.7% | 0.850 | 70.0% | 100.0% | 0.937 | 100.0% | 86.8% |
| hsa-miR-320a + hsa-miR-6515-5p | 0.996 | 93.3% | 98.2% | 0.999 | 72.0% | 100.0% | 0.969 | 99.1% | 86.0% |
| hsa-miR-1260b + hsa-miR-6515-5p | 0.970 | 96.7% | 83.6% | 0.999 | 74.0% | 100.0% | 0.980 | 99.1% | 81.8% |
| hsa-miR-320a + hsa-miR-1260b + hsa-miR-6515-5p | 0.937 | 96.7% | 89.1% | 0.880 | 74.0% | 100.0% | 0.942 | 100.0% | 86.7% |

**Supplementary Figure 3.**Classifiers trained by logistic regression using selected miRNAs and their different combinations were not superior than classifiers simply using cut-off of a single miRNA.

| **Table 4Clinical information of GSE124158, GSE113486, GSE106817and GSE112264** | | | | | | | | |
| --- | --- | --- | --- | --- | --- | --- | --- | --- |
|  | **GSE124158** | | **GSE113486** | | **GSE112264** | | **GSE106817** | |
|  | Healthy control | Gastric cancer | Healthy control | Gastric cancer | Healthy control | Gastric cancer | Healthy control | Gastric cancer |
| Total | 274 | 30 | 100 | 40 | 41 | 50 | 2759 | 115 |
| Sex | Female: 125  Male: 149 | uncertain | Female: 52  Male: 48 | Female: 9  Male: 31 | Female: 0  Male: 41 | Female: 0  Male: 50 | uncertain | uncertain |
| Age | 51.19 ± 12.18 | uncertain | 64.35 ± 15.57 | 65.15 ± 10.53 | 63.22 ± 17.03 | 67.94 ± 9.35 | uncertain | uncertain |
| Tumor stage | Not Available | uncertain | Not Available | uncertain | Not Available | uncertain | Not Available | uncertain |

**Supplementary Figure 4.**Clinical information of the for datasets we selected and all the information were extracted from phenoData in corresponding GEO datasets.
